# The Development of Immunotherapy Strategies for the Treatment of Type 1 Diabetes

**DOI:** 10.3389/fmed.2018.00283

**Published:** 2018-10-09

**Authors:** Ken Coppieters, Matthias von Herrath

**Affiliations:** Novo Nordisk (Denmark), Copenhagen, Denmark

**Keywords:** type 1 diabets, immunotherapy of cancer, insulin, tolerance, trials

## Abstract

Optimized insulin therapies, increased use of continuous glucose monitoring/insulin pumps and most importantly the arrival of reliable closed loop systems will undeniably lead to a reduction in the burden of complications that arise from type 1 diabetes. However, insulin therapy will only ever treat the symptoms of the disease and will not alter the underlying pathology. The aim of immunotherapy treatment is to modulate the immune system, a strategy that has been successful in autoimmune conditions such as multiple sclerosis, rheumatoid arthritis and lupus. However, the success rate of immunotherapy treatment in type 1 diabetes has been low. There are several distinct stages of T1D development. In this review, we summarize the most important immunotherapeutic approaches tested thus far and focus on the characteristic features and unmet need within the different stages of the disease.

## Rationale for immunotherapy in T1D

### Background-T1D as an autoimmune disorder

Type 1 diabetes is characterized by the progressive loss of pancreatic beta cell function, eventually culminating in patients' dependence upon exogenous insulin to control blood glucose. Despite continuing improvements in insulin therapy, the majority of patients fail to adequately control their glucose homeostasis (1), resulting in both short-term (hypoglycemia) and long term (nephropathy, retinopathy, neuropathy, and others) complications. This well-documented fact leads to a significant unmet medical need and there is therefore an incentive to develop disease modifying therapies which could be used in addition to symptomatic treatments.

Disease modifying therapies by definition are designed to tackle the underlying cause of the condition. It has been known for decades that T1D is associated with autoantibodies (2) and inflammatory infiltration of the pancreatic islets (3). Early genetic evidence revealed a profound contribution of the HLA region, (and MHC class II in particular). With the advent of the GWAS era, many of the susceptibility regions were shown to code for proteins important in immune function and considerable overlap was found with the genetic signature of other autoimmune conditions (4). The combined evidence thus overwhelmingly favors a pivotal role of leukocytes, and especially T cells, during beta cell destruction.

The T cell repertoire associated with T1D development is by all accounts diverse and is directed against beta cell specific molecules such as insulin, as well as autoantigens that are also expressed in other tissues, such as GAD. Although some progress has been made in using T cell signatures as disease biomarkers in individual patients (5), the kinetics and composition of these repertoires still appear to be largely unpredictable.One of the reasons may be that these parameters are typically measured in blood samples without the possibility of linking any observations to events at the target organ, which is extremely difficult to biopsy.

Finally, the autoimmune response and accompanying beta cell decay should be seen as a chronic, subclinical process that is initiated by unknown environmental factors long before clinical diagnosis. What has been unequivocally established is that autoantibodies serve as a reliable predictor of disease. Subjects with multiple autoantibody species carry a lifetime risk of developing T1D approaching 100% (6). This is a very powerful measure and it could be that high risk of T1D should be treated as a distinct, yet silent indication (6); much like hypertension is defined as a prodrome to stroke and myocardial infarction (7).

### Immunotherapy from a patient perspective

Before looking into any potential immunotherapy targets, careful consideration should be given to the desired clinical outcomes. First and foremost, T1D presently is considered a chronic metabolic condition which can be adequately controlled with modern insulin therapy. Any disease modifying therapy administered at any given disease stage should therefore be exquisitely safe. This therefore excludes chronic immunosuppression regimens, which may have a safety/efficacy balance that is acceptable in situations of high risk (for instance transplantation), but carry side effects related to host defense and tumorogenicity that are unacceptable in T1D.

The clinically relevant efficacy outcomes for immunotherapy in T1D depend on the disease stage being treated. A consensus paper was recently published that defines four distinct stages of T1D development, three of which are situated prior to conventional diagnosis and one after (6). A close look at the characteristic features and unmet need within each of these stages helps to optimally frame experimental animal work and data interpretation.

#### Pre-stage 1: genetic susceptibility and genetic risk

At this stage individuals carrying T1D susceptibility alleles have not yet developed islet autoantibodies. The risk/benefit proposition to incentivize patients, physicians and payers to commence preventive therapy at this stage will depend on the individual degree of risk, which for instance in case of multiple affected first degree relatives only amounts to 20–25% (8). Furthermore, the progression rates vary wildly, with many years' difference in time of onset even between identical twins (9). As an example of screening efficiency, the German Fr1da study tested ~27,000 children aged 2–5 years for antibodies at routine pediatric health exam visits and ended up with ~0.4% harboring antibodies (10). This renders clinical development for this sub-indication a lengthy and costly endeavor, also taking into account that most subjects in this segment will be pediatric cases.

All the above implies that immunotherapy at this stage should be superiorly safe, convenient, and efficacious in delaying clinical diagnosis. On the other hand, this is arguably the stage that from a mechanistic immune modification/suppression perspective, treatment with immunotherapy would be most likely to be successful. Since no signs of active autoimmunity are present, one could argue that the autoreactive T cell repertoire has not expanded and adopted a memory phenotype, a state which many believe is hard to reverse. This notion is also supported by the vast majority of animal models studies, with many experimental therapies proving efficacious only when administered to neonatal or juvenile animals. We argue that some of the failures in clinical translation may stem from the inappropriate extrapolation of pre-clinical data situated within the animal model equivalent of Pre-Stage 1 into trial designs including exclusively Stage 3 patients. Antigen-specific therapies could be well suited for this stage and will be discussed below for the example of oral insulin.

#### Stage 1: autoimmunity+ normoglycemia (presymptomatic)

This is the stage where individuals have developed measurable signs of autoimmunity in the form of autoantibodies. It can be inferred that some time before, seroconversion processes such as islet antigen presentation, T cell activation and plasma cell formation have taken place and that insulitis has been initiated in some parts of the pancreas. However, the histopathological experience from nPOD, the largest collaborative tissue database for T1D, indicates that very limited beta cell loss occurs prior to diagnosis (11). This may suggest that the bulk of immunological destruction occurs around diagnosis in response to a putative environmental trigger, such as a viral infection. Indeed, a study measuring enterovirus RNA or viral protein in blood, stool, or tissue of patients with pre-diabetes and diabetes found that there was a clinically significant association between enterovirus infection and T1D (12). Recent data support two clear phases of C-peptide decline following this initial event: an initial exponential fall over a 7-year period, followed by a prolonged stabilization where C-peptide levels no longer decline (13).

When multiple islet autoantibodies are detected, patients, physicians, and payers can be presented with the prospect of a lifetime risk approaching 100%. The antibody assays are currently used at the time of diagnosis but a consortium consisting of academic and industry partners led by the Critical Path Institute (https://c-path.org/) is currently working toward regulatory approval of these biomarkers for use in development programs in the presymptomatic stages of T1D.

This stage, along with Stage 2, can be experimentally modeled by including animals at a later age when the disease process is already advanced and the autoreactive T cell repertoire to some extent has expanded and adopted a memory phenotype. Whereas antigen-specific therapy would fulfill the requirements pertaining to safety, the thought is emerging that conventional antigenic tolerization strategies may be insufficient to tolerize T cells already committed to a functional activated memory lineage.

#### Stage 2: autoimmunity+ dysglycemia (presymptomatic)

Much like in Pre-Stage 1, clinical development in Stage 1 is complicated by the long and varying progression rate to diagnosis, with 5-year and 10-year risks being ~44 and 70%, respectively. By implementing glucose tolerance testing, 5-year risk can be increased to ~75%, which reduces both trial size and duration (6). An attractive proposition for both Stage 1 and 2 individuals would be to reduce disease risk by half or double the time to diagnosis. Other than treating as late as possible prior to hyperglycemia development, Stage 2 is not routinely separated from Stage 1 in animal models.

#### Stage 3: autoimmunity+ dysglycemia (symptomatic)

By far the majority of development activity for immunotherapies in T1D has focused on this disease stage, immediately after diagnosis. At that point, patients are assumed to have lost up to 90% of their functional beta cell mass, yet many retain a fraction accounting for measurable C-peptide levels. The DCCT trial long ago indicated that patients benefit from this preserved endogenous insulin secretion through reduced risk for long and short term complications (14). It has therefore been proposed that immunotherapy, while not sufficient to restore normoglycemia, could be employed to preserve remaining beta cell function at clinical diagnosis.

The onset of clinical symptoms and the consequent need for exogenous insulin therapy result in a slightly less sensitive risk/benefit balance as compared with the presymptomatic stages. Since the disease has progressed to full-blown islet destruction driven by a fully activated autoreactive repertoire, antigen-specific monotherapy no longer is a viable option. To our knowledge, not a single antigen-specific therapy is able to reverse hyperglycemia in autoimmune diabetes models. The prevailing view is that the disease should at this stage be modified with a short course of pathway specific immune modulators such as biologicals, alone or preferably in combination with antigen-specific maintenance therapy.

From a value perspective, immunomodulatory strategies at this stage have come under pressure in recent years. The prognosis is that optimized insulin therapies, increased use of continuous glucose monitoring and insulin pumps and most importantly the arrival of reliable closed loop systems will reduce the burden of complications in T1D. The added value of maintaining endogenous C-peptide at the expense of diminished immune function, even if temporary, thus becomes less attractive. Nonetheless, owing to the more easily available patient population, this stage could in the future be utilized to obtain more rapid proof-of-concept results prior to embarking on more resource-draining prevention studies.

#### Disease heterogeneity

Before transitioning to a discussion of some of the therapeutic concepts and studies situated in the 3 disease stages outlined above, the heterogeneous natural history of T1D deserves highlighting. It is clear that both rate of progression to clinical onset in the prevention stage and loss of C-peptide after onset show high inter-individual variation. Some underlying variables are well known such as the relationship between age at onset and rate of C-peptide decline. The majority of mechanistic factors underlying variability in disease course, however, remain poorly characterized.

This heterogeneity considerably affects trial size. For instance, while individuals with multiple autoantibodies have a near 100% lifetime disease risk, the 3- and 5-year risk which is relevant to outcome trials, happens to be much lower. The community has attempted to address this problem for instance by seeking to identify fast progressors via more comprehensive risk scores (15) or in stage 3 by correlating immune biomarkers with metabolic outcomes (16). This has proven to be extremely difficult in a polygenic autoimmune disease such as T1D, with hyperglycemia onset being the likely consequence of diverging immunopathological pathways.

Finally, biomarkers that are able to predict therapeutic responders would be the first step toward the holy grail of personalized medicine. Numerous attempts have been made with mostly some interesting *post-hoc* responder correlation findings (17, 18). We are, however, not aware of studies that managed to identify solid response biomarkers that would support use as an inclusion criterion for further studies. The market reality for industry is also such that, unless the target population can be cost-effectively identified with exquisite specificity and sensitivity, the business case for sub fractionation of an orphan indication such as recent-onset T1D becomes rather difficult and fragile. On the positive side, it is our belief that the well characterized prognostic value of islet autoantibodies calls for moving toward development of drugs into this space and eventually population wide risk screening (6).

### A look at the present development landscape

Without intending to provide a complete overview [which can be found in Coppieters et al. (19)], we will discuss some of the more notable immunotherapies tested in T1D. Likely due to past failures and the limited financial case associated with treatment of a subgroup of T1D patients, few immunotherapy agents have been designed specifically for the treatment of T1D. Instead, most of the drugs tested in T1D have been repurposed from major autoimmune indications or the transplantation field.

#### Studies in the presymptomatic phase of T1D

Only a handful of trial consortia have consistently screened for and identified at-risk subjects, and therefore trial activities in the presympromatic stages have been relatively scarce. The most important consortium active in this space has been TrialNet (https://www.trialnet.org/), a US-based international clinical consortium that offers screening and trial inclusion. TrialNet has since its inception screened in excess of 160,000 subjects at a rate of ~15,000/year.

The case of oral insulin tolerization will be discussed in detail in the next section. Two ongoing TrialNet prevention studies using biologicals are of interest. Abatacept, a CTLA-4Ig fusion molecule approved in several autoimmune indications, had been trialed earlier in Stage 3 patients (20). A delay in C-peptide decline was observed exclusively in the first 6 months after treatment initiation and the effect disappeared during subsequent dosing. Considering that CTLA-4Ig acts through costimulation blockade, as part of the early T cell activation process, it could be argued that such priming events predominantly take place earlier in the disease process. Based on this assumption, a prevention trial is currently enrolling Stage 1 subjects (NCT01773707). However, CTLA-4 is expressed on the membrane of both conventional activated T cells and regulatory T cells, and so it is possible that using this approach in isolation, will not be successful.

One of those few dedicated T1D drugs with an elaborate pre-clinical and clinical history is the anti-CD3 monoclonal teplizumab. Once seen as the most promising immune modulator for T1D, it infamously failed in phase 3 trials in Stage 3 T1D (21, 22). Only treatment using the highest dose of teplizumab (and in particular in those randomized <6 weeks after diagnosis) led to preserved β-cell function for several months, maintaining significantly higher levels of C-peptide and allowing glycemic control to be achieved at a lower insulin dose in the teplizumab groups than in the placebo group. The somewhat contested composite endpoint of insulin usage and HbA1c (23) illustrates the issue raised above on attaining clinically relevant value, namely that an attractive and commercially viable product needs to offer more than C-peptide preservation. Anyhow, not unlike the rationale behind abatacept, it was reasoned that a course of T cell depletion earlier in the disease process may confer more meaningful benefit and an ongoing trial therefore targets Stage 2 T1D (NCT01030861).

Both the abatacept and teplizumab trials are expected to inform the R&D community on three key aspects of T1D drug development. First, one could question if potent T cell modulation or depletion with a proven drug does not delay the disease course, which type of T cell immunotherapy will? Second, if these trials succeed they validate the aforementioned strategy to use Stage 3 trial data as a gatekeeper for development in the pre-symptomatic phase. Lastly, positive data would validate the extensive pre-clinical datasets predicting the efficacy of T cell modulation, while negative data would cast doubt on their value.

A final trial worth mentioning is situated in the antigen-specific class. Diamyd® is a GAD-based vaccine formulated in alum adjuvant that previously failed to preserve C-peptide in Stage 3 patients during phase 3 development (24). The DIAPREV-IT trial was the first prevention study with Diamyd® and the results have been presented at ADA-2017 *(http://www.diabetes.org/newsroom/press-releases/2017/larsson-scientific-sessions-2017.html)*. The trial enrolled subjects at Stage 1 and 2, who received 2 subcutaneous doses. 18 out of 50 subjects developed T1D in the observation period with no significant differences between treated and placebo and no effect on C-peptide or blood glucose. Newly published pre-clinical data also appear to question the potential of GAD based vaccination strategies (25).

#### Studies in the symptomatic phase of T1D

As outlined above, this is the most accessible stage of disease from a trial recruitment perspective and most of the clinical development activity in immunotherapies has occurred in this space. TrialNet and another public clinical trial consortium, the Immune Tolerance Network (ITN), have performed many of the pioneering studies. Almost all drugs tested had been approved in other autoimmune or transplantation indications and taken into T1D studies based on varying degrees of evidence for overlapping disease pathways. Examples include rituximab [anti-CD20 (26)], abatacept [CTLA-4Ig (20)], alefacept [anti-CD2 (27)], canakinumab [anti-IL-1 (28)], and anti-thymoglobulin (ATG, pan-T cell (27)].

The results using imatinib (Gleevec), a tyrosine-kinase inhibitor approved for chemotherapy in cancer indications, in Stage 3 T1D were just presented at ADA-2017. C-peptide levels were significantly preserved vs. placebo and reduced exogenous insulin usage was accomplished at the expense of mild to moderate AEs (infection, gastrointestinal,…).

Dedicated T1D agents, such as the anti-CD3 monoclonals teplizimab and otilixizumab (29, 30), as well as GAD-alum (Diamyd) have shown great promise in terms of C-peptide preservation in phase 2 trials but failed to meet endpoints in phase 3 development.

Collectively, it can be concluded from the moderate and transient C-peptide preservation observed in some of the above trials that immunotherapy is indeed capable of disease modification as late as in Stage 3. However, different study designs, and testing sequential or repeated treatment may be advised to improve efficacy.

An alternative strategy that has gained traction is to target complementary pathways through combination therapy. Low-dose proleukin (IL-2)+rapamycin (31) and daclizumab (anti-CD25)+mycophenylate (32) were combination therapy examples, with the former actually showing temporary disease acceleration. Thus, increasing efficacy by interfering with distinct immune functions does not necessarily result in improved safety and tolerability, or trial complexity for that matter. A more recent study exploring the combination angle was ATG+ Neulasta (G-CSF) which demonstrated beta cell preservation in Stage 3 patients (33, 34). In a way, the polyclonal Treg cell transfer technology currently tested by Caladrius (NCT02691247) in itself is also an example of combination therapy since expected to target multiple disease pathways downstream of the Treg.

A special category of combination therapy includes both an immunologic agent and one that acts to preserve beta cell health/function (35). The rationale behind such an immune-metabolic combination is that tackling the immune component of the disease with a cocktail of immune modifiers alone often comes at the expense of side effects related to immune suppression. Furthermore, even if the autoimmune part of the disease is adequately addressed, survival and functionality of the remaining beta cell pool may need to be targeted from a distinct therapeutic angle. One such example may consist of an immune modifier in combination with a GLP-1R agonist, a peptide drug class commonly prescribed in T2D. Several studies have suggested that GLP-1R agonism has protective effects on the beta cells, likely through mechanisms of ER stress relief (36, 37). The hypothesis then is that simultaneously dampening the autoimmune component with an immune modifier and relieving beta cell stress could lead to improved beta cell survival and functionality. A Novo Nordisk study using a neutralizing anti-IL-21 antibody in combination with the GLP-1R agonist liraglutide is underway (NCT02443155) (38).

Finally, significant progress has been made in recent years on the generation of stem cell derived beta cells and their implantation to replace lost beta cell mass. San Diego-based Viacyte has now conducted the first phase 1 trial on the concept (NCT02239354) and hopes are high that longstanding patients and especially “brittle” diabetic cases will benefit from this approach. However, depending on the success of accompanying encapsulation devices being able to protect the grafts from allo-rejection and autoimmunity, an effective, tolerable, and safe immunotherapy may actually also be needed in this niche.

## Case in point: oral insulin tolerization

The concept of tolerization of the immune system through ingestion of antigenic substance dates back to ancient times. In the area of hypersensitivity and food allergies, the concept recently showed considerable promise with examples including protection against peanut (39) and egg white allergy (40). Within the context of autoimmunity, data from pre-clinical models and small proof-of-principle trials had suggested disease modifying action, which fueled larger scale trials. The company Autoimmune Inc., spun off from the results of Weiner and colleagues, tested oral tolerance therapy in major indications such as RA and MS but was ultimately unable to demonstrate significant disease amelioration (41).

A high profile endeavor in T1D was the clinical testing of oral insulin administration in at-risk subjects by TrialNet. The Diabetes Prevention Trial—Type 1 Diabetes (DPT-1) was the first major prevention trial with mass risk screening of relatives of T1D patients (42). Over 100.000 relatives were screened for islet autoantibodies and 372 were assigned to receive 7.5 mg /day oral insulin or placebo. At endpoint, the annualized rate of diabetes was similar in both groups. *Post-hoc* analysis did suggest that there was benefit in a subgroup with insulin autoantibodies (IAA), which formed the premise for a subsequent study in this population (43). Stage 1 participants with normal FPIR (first-phase insulin response) showed no delay or prevention. In a small subgroup (27 treated vs. placebo) with abnormal FPIR (=lower functioning beta-cells), oral insulin delayed T1D onset by an average of 31 months. The biological foundation for this observation remains unclear but it may point toward underlying heterogeneity of the disease.

Inspired by this long development history, we at Novo Nordisk recently concluded a careful experimental reassessment of the pre-clinical dataset on oral insulin in T1D (44). We first reasoned that timing of administration and dose are the most likely major variables that influence outcome. Considering the low-mg range doses typically given in mice, the 7.5 mg daily dose used in the DPT-1 trial does not represent the expected extrapolated dose going from animals to men.

Furthermore, many studies, including the original study by Weiner and colleagues, initiated treatment at 5 weeks of age in NOD mice (45). This age models Pre-stage 1 and we therefore found it important to assess disease prevention at 9 weeks of age, which would be the equivalent of Stage 1 as enrolled in DPT-1. Additional variables tested based on literature evidence were species origin of the insulin (46) and introduction of amino acid substitutions that rendered insulin metabolically inactive (47).

The sobering outcome was that none of the regimens tested in this treatment matrix resulted in disease protection (44). We found that orally administered insulin is degraded within minutes, which would also have been the case with the administration route used in DPT-1. A remarkable feature of gavaging insulin in large buffer volumes in mice was that the dosed solution travels immediately past the stomach into the small intestine, the purported site of action for oral tolerance induction. We therefore performed tolerance studies using endoscopic dosing of insulin in enteroprotective capsules in pigs but were unable to demonstrate any tolerizing effect (unpublished data).

Our negative oral insulin findings do not stand in isolation within the field of antigenic therapies for T1D. In collaboration with the Lenardo lab, we found no support for the tolerizing effect of parenteral, metabolically inactive insulin as had previously been reported (48). Likewise, published data on disease prevention using a strong agonist insulin mimetope did not appear to be reproducible (49).

What could be the reasons for these failures to reproduce pre-clinical data? Whereas the argument on animal colonies differing in terms of microbiome and disease penetrance might have basic scientific merit, it bears little relevance in view of the fact that therapies ultimately have to prove their value in an outbred human population within an uncontrolled environment. In other words, preclinical evidence should be robust enough to hold up in different vivariums. A possibility is that antigenic tolerance in general confers some degree of protection but is overall not potent enough to be universally reproducible. Thus, the labs where disease progression occurs less aggressively would be the ones observing benefits. For instance the original Weiner study had only 50% incidence in the control group, whereas we consistently reached around 70%.

Finally, it might be that, from a mechanistic point of view, antigen-specific monotherapy is unable to curb the established effector memory T cell responses that are characteristic for autoimmunity. The autoimmune response also qualitatively differs from the allergic response and that may be the reason why only the latter can still be modified late in the disease process. For T1D prevention, that is also what animal models have historically showed, namely that antigenic monotherapy only works in the very early disease stages equivalent to Pre-Stage 1. This hypothesis formed the rationale for the PRE-POINT study, which dosed Pre-Stage-1 in genetically at-risk, autoantibody negative children with oral insulin (max dose 67.5 mg) (50). Some immunological modification was observed upon dosing, and studies such as the Bavarian Fr1da insulin intervention study could elucidate whether this actually translates to prevention of seroconversion (NCT02620072). Rather than further narrowing down potential responder populations in later disease stages, we believe the Pre-stage 1 indication is the more applicable one going forward also based on the available body of animal model data.

## Conclusion

Immunotherapy for T1D has a checkered clinical history with a number of high-profile failures in the late development phase. In response, some have questioned the predictive value of animal models. We believe animal models continue to have their place in immunotherapy development for T1D, provided that they are used appropriately.

The current trend is toward combining drugs to enhance efficacy. An example would be Novo Nordisk's development program on anti-IL-21 program, where the original aim was to provide pre-clinical data in support of targeting a recent-onset T1D indication. While anti-IL-21 monotherapy potently prevents diabetes in the NOD model, it does not reverse.It was therefore opted to combine the GLP-1R agonist liraglutide with anti-IL-21, resulting in reversal after hyperglycemia onset in the NOD model. The program is currently in phase 2 in adult, recently diagnosed T1D patients, with primary endpoint on beta cell preservation (NCT02443155).

In conclusion, the past few decades have taught us that immunotherapy holds promise in T1D, but we haven't cracked the code yet in terms of acceptable safety/efficacy balance. We now have the knowledge to identify subjects earlier in the disease process before diagnosis, a disease state that might be easier to modulate. The near future will tell whether that hypothesis holds true, which would effectively turn T1D into a preventable condition.

## Author contributions

All authors listed have made a substantial, direct and intellectual contribution to the work, and approved it for publication.

### Conflict of interest statement

Both authors are employed by Novo Nordisk.
